# Neck Hair-Thread Tourniquet Syndrome by Co-sleeping With Family: A Case Report and Literature Review

**DOI:** 10.7759/cureus.39620

**Published:** 2023-05-28

**Authors:** Yuki Murata, Hiroshi Sakakibara

**Affiliations:** 1 Department of Pediatrics, Tokyo Metropolitan Children’s Medical Center, Tokyo, JPN; 2 Department of Pediatrics, Tokyo Metropolitan Children's Medical Center, Tokyo, JPN

**Keywords:** bed sharing, accidental strangulation, co-sleeping, near strangulation, hair tourniquet syndrome

## Abstract

Neck hair-thread tourniquet syndrome (NHTTS) is a rare condition that can be a pediatric emergency, occurring when a hair or thread becomes tightly wrapped around a body part, leading to vascular or tissue damage. NHTTS commonly affects infants and young children and can result in severe complications if not promptly diagnosed and treated. The unusual nature of this event, the diffuse petechial hemorrhage on the face, and the presentation of ligature marks extending around the neck led us to admit the child to the general pediatric ward for follow-up and further investigation of the possibility of non-accidental trauma.

Co-sleeping is a common cultural practice in Japan where parents sleep in close proximity to their infants. This case report aims to raise awareness among pediatricians and parents about the possibility of NHTTS occurring in infants who co-sleep, particularly when a strand of hair becomes entangled around their neck, about the early detection and appropriate management of NHTTS. And we also summarize the reported NHTTS cases.

## Introduction

Neck hair-thread tourniquet syndrome (NHTTS) is a rare condition that is an important emergency condition that requires urgent attention. It has been recognized since the 17th century [1,2]. Commonly affected sites include fingers, toes, or even genitals [1,3]. We report a case of NHTTS that was successfully released without complications. This report aims to emphasize the possibility of NHTTS occurring in Japan due to the widespread culture of co-sleeping, particularly when hair becomes entangled around an infant's neck during sleep, and to raise awareness among healthcare professionals and the general public about the early detection and appropriate management of NHTTS.

## Case presentation

**Figure 1:**
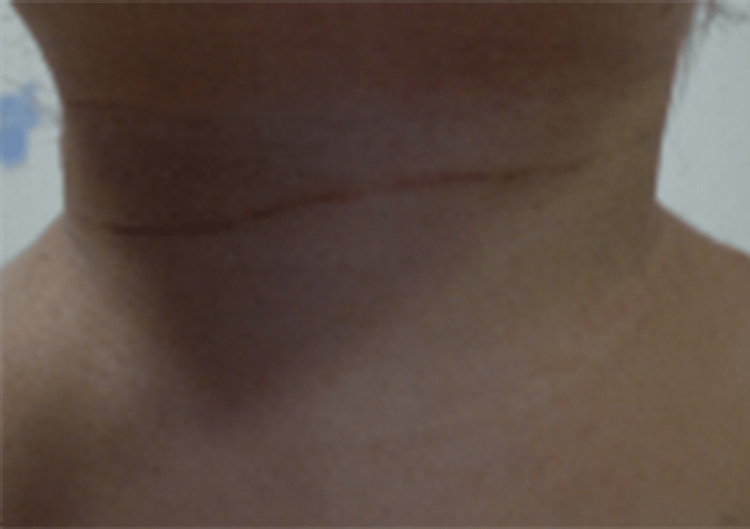
Photograph demonstrating traces of cervical strangulation.

**Figure 2:**
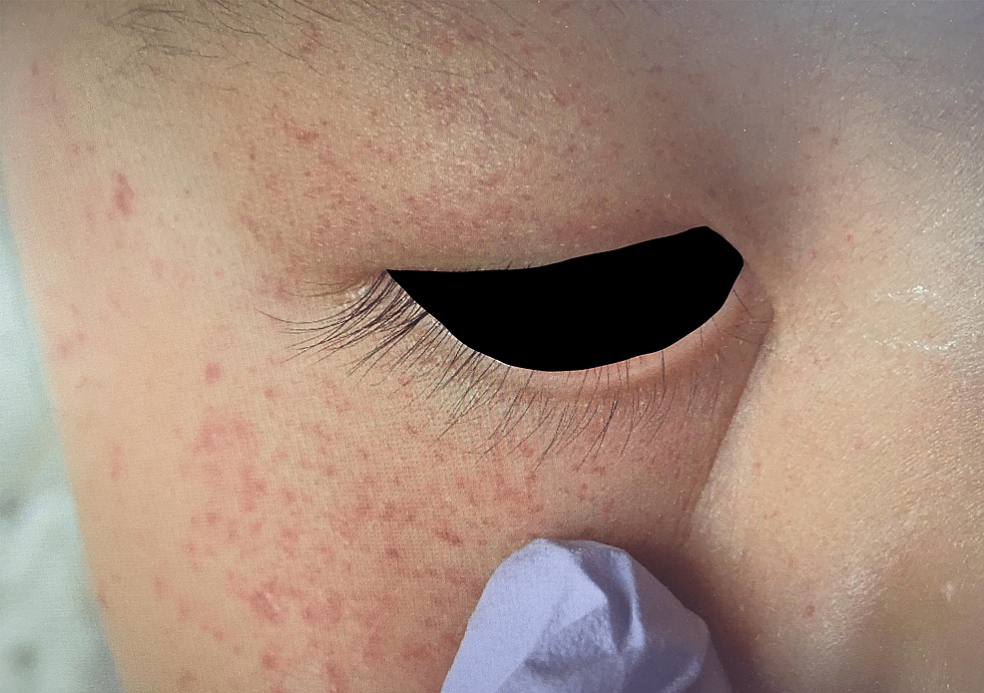
Photograph demonstrating more detailed periocular petechial hemorrhage.

A two-year-old boy was brought to the emergency department (ER) by his parents with complaints of irritability, crying, and swelling in the neck region. The parents reported that his mother, the boy, and his seven-year-old sister were sleeping next to each other and that the next day morning, the boy cried, and his sister's hair was found wrapped around his neck. The child had become increasingly agitated, and his mother unraveled and unlocked it by hand immediately, without cutting the hair. On physical examination, the child was found to have a hair-thread tourniquet encircling his neck, with moderate swelling and petechial hemorrhage in the periocular area (Figures 1, 2). The hair was tightly wound around the neck, causing compression of the underlying tissues and blood vessels. There was no evidence of respiratory distress, cyanosis, or neurological deficits.

Management

**Figure 3:**
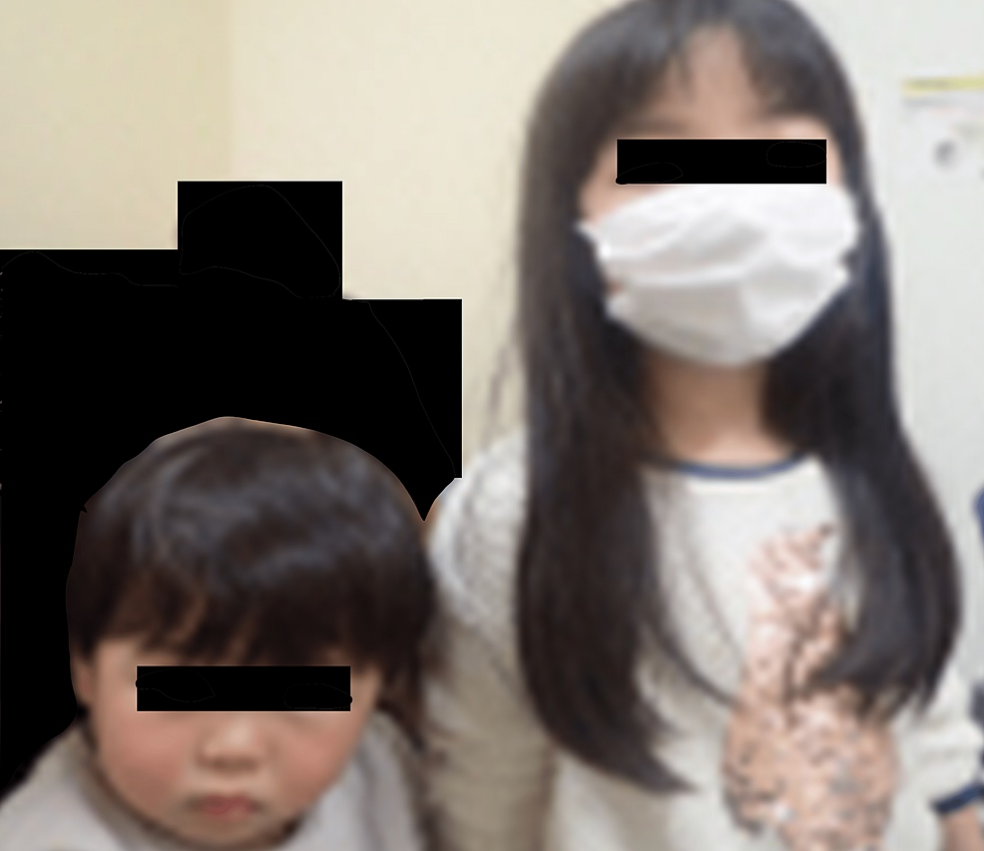
Photograph demonstrating his sister's long hair strangled his neck.

The child was promptly admitted to the hospital. The symptoms were immediately relieved, and the swelling and erythema gradually subsided over the next few days. The child was observed for 48 hours for any signs of complications, such as infection, bleeding, or tissue necrosis, but none were noted. Additional interviews revealed that the sister and brother get along well, the house was tidy, and nothing to point out during the social worker's visit. His sister's hair was long enough to curl around his neck (Figure 3). No other risk factors were noted in their social history. To prevent future accidents, we asked his sister to discuss with her family whether she would prefer to sleep with her hair tied or in a separate room. She decided to sleep in a different room to ensure safety.

## Discussion

NHTTS is a rare but potentially serious condition that requires prompt recognition and management. Hair-thread tourniquet syndrome (HTTS) is commonly seen in infants and young children, and hair, thread, or other fibrous materials are usually the cause [1]. The causes of HTTS were attributed to hair-related factors in 85.2% of cases, thread-related factors in 13.8% of cases, and both factors in 1% of cases [4]. The tourniquet effect can lead to tissue ischemia, edema, and necrosis and can even cause airway obstruction and death [5] in severe cases. To our knowledge, only six such cases of strangulation by human hair have been reported in the past 30 years caused by NHTTS when young children sleep with a family member with long hair [2,5-9]. Past reports indicate that out of the total of 210 reported cases, neck cases made up 0.95% of the total [4].

In this case, the child had a hair-thread tourniquet around the neck due to co-sleeping with family members. The diagnosis was made based on the clinical presentation. The child had an uneventful recovery and was discharged home in good condition.

HTTS is a highly urgent condition, and especially neck hair tourniquet can pose a high risk to life, with co-sleeping with a mother or sibling being one of the causes [2,5-9]. Bed sharing is a controversial topic in the US due to its association with the risk of sudden infant death syndrome (SIDS). According to previous studies, bed-sharing is more common among Asian families than white families [10,11]. However, there is also evidence that bed-sharing is increasing regardless of race. As bed-sharing is recognized as a risk factor for SIDS [12], organizations such as the American Academy of Pediatrics (AAP) are working to raise awareness of the dangers of bed-sharing [11,13].

On the other hand, there are also reports that bed-sharing itself can have positive effects on psychological stability and bonding with family members. Bed sharing is correlated with breastfeeding [14]. Ward conducted a systematic literature review on mother-infant bedsharing and identified 10 reasons why mothers choose to bedshare including (1) breastfeeding, (2) comfort for mother or infant, (3) improved/more sleep for infant or parent, (4) monitoring, (5) bonding/attachment, (6) environmental factors, (7) crying, (8) cultural or familial traditions, (9) disagreement with danger, and (10) maternal instinct (Table 1) [15].

**Table 1:** Features of co-sleeping

Benefits	
Advantages	breastfeeding, comfort for mother or infant, improved/more sleep for infant or parent, monitoring, bonding/attachment, environmental factors, crying, cultural or familial traditions, disagreement with danger, maternal instinct
Disadvantages	NHTTS, SIDS, falling from bed, asphyxiation

Bed-sharing has both advantages and disadvantages, and it cannot be completely prohibited. Therefore, we believe that it is difficult to prohibit sleeping together completely due to cultural aspects. Using Japan as an example, Japanese children have traditionally slept with parents, especially mothers, in physical proximity. “Soine (Japanese for co-sleeping)” means that a mother and a baby sleep together on the same futon (quilted Japanese-style mattress laid out on the floor) next to each other.

We conducted a literature review of changes over time in co-sleeping. In the early 1960s, 90% of three- to four-month-old infants slept with their mothers in the same room [16]. Co-sleeping was as common among Japanese mothers in 2008-2009 as it had been in the 1960s and 1980s. In the 2008-2009 survey, 72% of mothers reported sleeping with their baby in the same room and within arm's reach [17]. From 2010 to 2012, mothers who co-slept with their infants made up 51.7% of participants [18]. Although the rate of co-sleeping appears to be declining with the westernization of lifestyle, another report shows that the rate of bed-sharing remains at 44.9%-56.3% up to the head control but increases to 76.2% at 10 months [19]. We think this may be attributed to the unique Japanese culture of putting newborns to sleep in cribs and transitioning to an old tradition of sleeping with them during infancy. 

We searched the literature to clarify common risk factors in the situations in which NHTTS occurred. Indeed, we found that all cases with co-sleeping. They have been reported in infants ranging from 11 months to 27 months (Table 2), including our case [2,5-9]. Furthermore, 85% of cases are with ligature marks on the neck and petechiae on the face. By country, there were four cases in the U.S., two in Japan, and one in the U.K.

**Table 2:** Summary of previously reported cases of NHTTS

Case	Ref No.	Age (month)	Sex	Cause	Consequence
1	2	27	male	mother's hair	non-accidental injury was ruled out
2	5	13	female	mother's hair	abuse was denied in a simulation with mannequins and patient's discharged home
3	6	19	male	sister's hair	hospitalization
4	7	11	female	sister's hair	discharged home with follow-up
5	8	19	male	mother's hair	non-accidental injury was ruled out
6	9	13	male	mother's hair	hospitalization, and discharged home, non-accidental injury was ruled out
7	our case	25	male	sister's hair	hospitalization, and discharged home with follow-up

From the above, the risk of NHTTS may increase during the period when infants begin sleeping with their parents or sister who have long hair. Six of the seven cases (85.7%) occurred in infants aged one year and older, and another case was 11 months old (Table 2). It is presumed that active movement during sleep and co-sleeping could result in NHTTS. It can happen in any country, especially in areas where co-sleeping is culturally well-accepted. The importance of public awareness should be emphasized. When we recognize a family that is sleeping with a mother or sister with long hair, we need to tell them that they are at risk for NHTTS and to prevent it.

The ER of Tokyo Metropolitan Children's Medical Center receives about 35,000 patients every year (20% of them trauma-related). And among the patients who visited our ER from March 2010 to December 2017, there were eight HTTS cases and no NHTTS cases [20]. Although it could be argued that some issues were released at home without visiting the ER, the threshold for ER visits is very low under the Japanese medical situation, where medical care for children under 15 years of age is essentially free. Therefore, our data would suggest NHTTS cases are not so common if not rare. It will be necessary to continue to accumulate cases and educate the public.

## Conclusions

NHTTS is a rare pediatric emergency and in all cases occurs while sleeping with a child is more likely to occur in infants over one year of age. It requires prompt recognition and management. Parents should be advised to prevent NHTTS by wearing their hair short if the family member has long hair when sleeping with their infant. Pediatricians and emergency physicians should be familiar with the clinical presentation of NHTTS and should consider it in the differential diagnosis of neck swelling and irritability in young children.
